# 96 Week Follow-Up of HIV-Infected Patients in Rescue with Raltegravir Plus Optimized Backbone Regimens: A Multicentre Italian Experience

**DOI:** 10.1371/journal.pone.0039222

**Published:** 2012-07-11

**Authors:** Amedeo Capetti, Simona Landonio, Paola Meraviglia, Antonio Di Biagio, Sergio Lo Caputo, Gaetana Sterrantino, Adriana Ammassari, Barbara Menzaghi, Marco Franzetti, Giuseppe Vittorio De Socio, Giovanni Pellicanò, Elena Mazzotta, Alessandro Soria, Marianna Meschiari, Michele Trezzi, Lolita Sasset, Benedetto Maurizio Celesia, Patrizia Zucchi, Sara Melzi, Elena Ricci, Giuliano Rizzardini

**Affiliations:** 1 1st Division of Infectious Diseases, “Luigi Sacco” Hospital, Milano, Italy; 2 2nd Division of Infectious Diseases, “Luigi Sacco” Hospital, Milano, Italy; 3 Infectious Diseases Clinic, “San Martino” Hospital, Genova, Italy; 4 Infectious Diseases Clinic, “Santa Maria Annunziata” Hospital, Firenze, Italy; 5 Division of Infectious Diseases, “Careggi” Hospital, Firenze, Italy; 6 3rd Division of Infectious Diseases, “Lazzaro Spallanzani” Hospital, Roma, Italy; 7 Division of Infectious Diseases, “Ospedale di Circolo”, Busto Arsizio, Italy; 8 3rd Division of Infectious Diseases, “Luigi Sacco” Hospital, Milano, Italy; 9 Division of Infectious Diseases, "Santa Maria della Misericordia" Hospital, Perugia, Italy; 10 Division of Infectious Diseases, “Policlinico G Martino”, Messina, Italy; 11 Division of Infectious Diseases, “Santo Spirito” Hospital, Pescara, Italy; 12 Infectious Diseases Clinic, “San Gerardo de’ Tintori” Hospital, Monza, Italy; 13 Infectious Diseases Clinic, Azienda Ospedaliera Universitaria Policlinico, Modena, Italy; 14 Division of Infectious Diseases, “Santa Maria della Misericordia” Hospital, Grosseto, Italy; 15 Division of Infectious Diseases, “Santa Maria della Misericordia” Hospital, Rovigo, Italy; 16 Infectious Diseases Unit, University of Catania, ARNAS (Azienda Ospedaliera di Rilievo Nazionale e di Alta Specializzazione) Garibaldi, Catania, Italy; Centro Nacional de Microbiología - Instituto de Salud Carlos III, Spain

## Abstract

**Background:**

Long term efficacy of raltegravir (RAL)-including regimens in highly pre-treated HIV-1-infected patients has been demonstrated in registration trials. However, few studies have assessed durability in routine clinical settings.

**Methods:**

Antiretroviral treatment-experienced patients initiating a RAL-containing salvage regimen were enrolled. Routine clinical and laboratory follow-up was performed at baseline, week 4, 12, and every 12 weeks thereafter. Data were censored at week 96.

**Results:**

Out of 320 patients enrolled, 292 (91.25%) subjects maintained their initial regimen for 96 weeks; 28 discontinued prematurely for various reasons: death (11), viral failure (8), adverse events (5), loss to follow-up (3), consent withdrawal (1). Eight among these 28 subjects maintained RAL but changed the accompanying drugs. The mean CD4+ T-cell increase at week 96 was 227/mm^3^; 273 out of 300 patients (91%), who were still receiving RAL at week 96, achieved viral suppression (HIV-1 RNA <50 copies/mL). When analyzing the immuno-virologic outcome according to the number of drugs used in the regimen, 2 (n = 45), 3 (n = 111), 4 (n = 124), or >4 (n = 40), CD4+ T-cell gain was similar across strata: +270, +214, +216, and +240 cells/mm^3^, respectively, as was the proportion of subjects with undetectable viral load. Laboratory abnormalities (elevation of liver enzymes, total cholesterol and triglycerides) were rare, ranging from 0.9 to 3.1%. The mean 96-week total cholesterol increase was 23.6 mg/dL.

**Conclusions:**

In a routine clinical setting, a RAL-based regimen allowed most patients in salvage therapy to achieve optimal viral suppression for at least 96 weeks, with relevant immunologic gain and very few adverse events.

## Introduction

The efficacy of raltegravir (RAL) in combination with optimized background therapy (OBT) in HIV-1 infected, treatment-experienced patients, was assessed in a multicenter, dose-ranging, randomized, placebo-controlled phase II study [1]. It was then confirmed in two randomized (2∶1), placebo-controlled phase-III international trials – BENCHMRK-I and -II – conducted in different geographic regions in a large population (n = 703) of HIV-1 infected patients failing therapy, with triple-class resistance mutations [2].

Apart from these sponsored studies, the amount of observational cohort data is relatively small. In the ANRS CO3 Aquitaine Cohort [3], 38/52 patients failing combination antiretroviral therapy (cART) had plasma HIV RNA <50 copies/mL at week 24 of a new RAL-based regimen, and frequent (9/11) integrase mutations in viral failures, mainly at positions 148 and 155. Another cohort of 36 subjects failing cART and 21switching for intolerance to RAL-based regimens with a mean genotypic sensitivity score (GSS)  = 2 [4], showed optimal viral response at week 48 in 87.7% with a mean immunologic gain of +75 CD4/mm^3^. Engsig et al. [5] compared 32 multi-experienced patients treated with RAL-based rescue therapy with 64 naive patients starting combination antiretroviral therapy (cART) and observed comparable immunological and virological outcomes over 72 weeks. Caby et al. reported the RAL-based rescue of 67 experienced patients harbouring multidrug resistant strains [6]. At week 24, 43 (64.2%) had HIV RNA <40 copies/mL, 18 had 40 - 400 (incomplete viral suppression, IVS) and 6 had >400 copies/mL. At week 48, two of the IVS group had overt failure, while the other 16 remained 40 – 400. Integrase mutations were detected in 6/8 overt viral failures. Having a GSS  = 0 for the backbone was predictive of viral load >40 (OR 20.9) and the development of resistance-associated mutations (RAMs), odds ratio (OR) 14.2. Another experience from Italy [7] prospectively evaluated 28 triple-class experienced patients harboring R5-tropic virus, treated with maraviroc, raltegravir and etravirine. At week 48, 26 (92%) had HIV-RNA <50 copies /mL. The authors reported 3 serious adverse events: one recurrence of mycobacterial spondylodiscitis, one anal cancer, and one Hodgkin lymphoma. The largest non sponsored study, the ANRS 139 TRIO, assessed a standard rescue regimen made of RAL, daryunavir and etravirine [8], in 103 HIV-infected subjects failing ART but harboring strains sensitive to all the study drugs. At week 48, 86% had an HIV RNA level <50 copies/mL. Grade 3 or 4 clinical adverse events were reported in 15 patients (14.6%).

As well as efficacy, also tolerability data are mainly obtained from the few official trials and describe a very well tolerated drug [9]. Rare reported adverse events related to raltegravir include acute renal failure correlated with rhabdomyolysis [10] and cerebellar ataxia [11]. Therefore we set up this observational multicentre study, to assess the effectiveness and tolerability of RAL-based regimens in multi-experienced, multi-failed patients requiring rescue therapy, starting from confirmed measurable viremia.

## Methods

### Patients

Treatment-experienced patients starting a raltegravir-including antiretroviral regimen between March, 2007 and June, 2009, were consecutively enrolled in a collaborative Italian study, called SALIR (SALvage in Italy with Raltegravir), having signed informed consent within the Expanded Access Program (EAP), approved by the local Ethics’ Committees, and followed beyond the time limit of the EAP, as convened among the authors, according to the Good Clinical Practice (GCP) Guidelines.

List of the Ethics’ Committees:

Comitato Etico Azienda Ospedaliera Luigi Sacco – Polo Universitario.

Comitato Etico delle Aziende Sanitarie dell’Umbria.

Comitato Etico Arnas Garibaldi.

Comitato Etico Irccs Ospedale San Martino di Genova.

Comitato Etico Provinciale di Modena.

Comitato Etico Sperimentazione Clinica Azienda Ospedaliera-Universitaria Careggi.

Comitato Etico Istituto Nazionale delle Malattie Infettive “Lazzaro Spallanzani”.

San Gerardo Hospital Ethics Committee.

Comitato Etico Ospedale di Circolo Di Busto Arsizio.

Comitato Etico per la Sperimentazione dei Farmaci della Ausl 9 di Grosseto.

Comitato Etico per la Sperimentazione Clinica dei Medicinali dell̀Azienda Sanitaria di Firenze.

Comitato Etico Scientifico A.O.U. Policlinico "G. Martino" – Messina.

Comitato Etico per la Regolamentazione della Sperimentazione Clinica dei Farmaci dell’Azienda U.S.L. di Pescara.

Comitato Etico Provinciale per la Sperimentazione Clinica dell̀Azienda ULSS 18 di Rovigo.

Companion drugs were chosen by each investigator on the basis of the genotypic sensitivity of the strains and of the patient’s history of treatment, intolerance, toxicity or allergy to antiretrovirals. All patients had blood drawn and visits at week 4 and 12 after the introduction of the new regimen, and approximately every 12 weeks thereafter. For the toxicity analysis we considered only those parameters that were routinely collected in all centers (alanine aminotransferase [ALT], aspartate aminotransferase [AST], total cholesterol [TC] and triglycerides [TG]) and considered the Common Terminology Criteria for Adverse Events (CTCAE, Version 4.03, June 14, 2010) [12]. Adverse clinical events and deaths were reported to the local Ethics Committees and authorities as defined by the law. Data were collected by the local investigators and periodically sent to the coordinating centre at “Luigi Sacco” Hospital, Milan. All data were censored at week 96 for homogeneity, however the investigators convened to continue the follow-up to allow further data in the future.

### Statistical Analysis

Baseline patients’ characteristics were described. Continuous variables were reported as means and standard deviation (SD) if they were normally distributed and as medians and interquartile range (IQR) if not. Ordinal and categorical variables were reported as frequency and percentage.

Patients who had missing data before week 96 were considered as failures. The proportion of subjects reaching <50 copies HIV-1 RNA/mL was analyzed as on-treatment (OT, the denominator being the population still on treatment at each time-point) and intention-to-treat: missing  =  failure (ITT:M = F, where the denominator is the number of subjects enrolled). Variables associated to treatment failure were compared using logistic regression, according to the Cox model. Results were given as hazard ratio (HR), and 95% confidence interval (CI). Odds ratio (OR) and 95% confidence intervals (CI) was used to evaluate the association between metabolic alterations at study end (missing patients were excluded from the analysis) and baseline patients’ characteristics. As appropriate, we included selected variables to adjust simultaneously for their potentially confounding effects, using a backward selection method and stating the significance cut-off P at 0.15.

## Results

**Figure 1:**
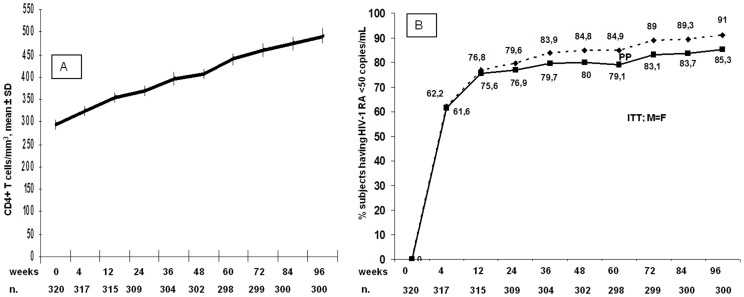
CD4+ T cells/mmc slope over 96 weeks and proportion of subjects achieving and maintaining optimal viral suppression, set for homogeneity at <50 copies/mL. The dimensions of the population at each timepoint is reported below (n). In figure 1A values are presented as mean±standard deviation (SD). In figure 1B the spotted line presents data in the on treatment analysis, while the continuous line shows the intention-to-treat, missing data  =  failure analysis (ITT; M = F).

Three hundred and twenty treatment-experienced patients have started a RAL-based regimen at least 96 weeks before the analysis, and of these 291(91.25%) were still receiving their baseline therapy at week 96. Demographic and immuno-virological characteristics of all the subjects are shown in Table 1. Twenty-eight (8.8%) patients discontinued prematurely their initial regimen. Five (1.6%) discontinued due to adverse events (1 for grade 3 rhabdomyolysis, 1 acute psychosis, 1 abdominal pain and 2 for fatigue and malaise), 3 were lost to follow-up, one withdrew consent, 8 (2.5%) experienced virologic failure and 11 (3,4%) died (5 of non-Hodgkin’s lymphoma, 3 of cirrhosis-related events, 1 of acute myocardial infarction, 1 of violent cause and 1 of tuberculosis). The mean immunologic increase by week 96 was +227 CD4+ T lymphocytes/mm^3^ (Figure 1).

**Table 1:** Main demographic features of the SALIR cohort (n = 320).

Age (years, mean ± SD)		47.3	8.7
Sex (n, %)	male	235	73.4
	female	85	26.6
HIV transmission category (n, %)	IDU	106	33.1
	Heterosexual	124	38.8
	MSM	68	24.4
	Other or unknown	12	3.8
CDC stage C (n, %)		144	45.0
CD4 count (cells/µL, n, %)	<200	143	44.8
	200–350	89	27.9
	>350	88	27.3
Past ARV regimens	1*–*5	72	22.5
	6*–*8	85	26.6
	9*–*12	94	29.4
	13	69	21.6
ART duration before study entry (years,mean ± SD)		16.9	5.5
Genotypic Sensitivity Score (mean ± SD)		2.0	0.8
HCV or HBV positive (n, %)		111	34.7
Total cholesterol (mean ± SD)		181	49
HDL cholesterol (mean ± SD)		46	25
Triglycerides (median, IQR)		170	118–275
AST (median, IQR)		32	22–48
ALT (median, IQR)		36	22–55

SD  =  Standard Deviation, IDU  =  intravenous drug user, MSM =  male having sex with males, IQR  =  interquartile range, ARV  =  antiretroviral, ART =  antiretroviral therapy, HDL  =  high density lipoprotein, AST  =  aspartate aminotransferase, ALT  =  alanine aminotransferase.

### Virologic Failure and Resistance-associated Mutations (RAMs)

By the week 96, 27 patients (8.4%) had detectable viremia, mainly due to adherence problems, as detected by pill count. Overall, 29 patients (8.9%) experienced early or late virologic failure.

Table 2 shows the results of logistic regression analyses (ITT and OT). At univariate analysis, stage C and CD4 cell count were significantly associated with failure in the ITT analysis; at the multivariate analysis, these factors stayed as the only predictors of failure, though CD4 cell count was borderline associated. None of the considered variables was significantly associated to failure in the OT analysis, though CDC stage C and CD4 cell count showed similar HRs as in the ITT. The adjusted HRs were similar but not statistically significant.

**Table 2:** Predictors of raltegravir effectiveness.

	ITT, n = 320, failure = 47			OT, n = 300, failure = 27	
	Crude HR(*95% CI*)	Adjusted HR*(*95% CI*)		Crude HR(*95% CI*)	Adjusted HR*(*95% CI*)
Age (by 5 years)	0.90 *(0.75–1.07)*			0.91 *(0.72–1.14)*	
Sex (Female vs Male)	1.00 *(0.52–1.92)*			1.22 *(0.54–2.79)*	
HIV transmission category (IDU as reference)					
Heterosexual	0.59 *(0.25–1.41)*			0.45 *(0.13–1.65)*	
Male Homosexual	1.16 *(0.62–2.17)*			1.33 (0.59*–*3.00)	
CDC stage C (Yes *vs* No)§	**2.37** *(1.30–4.33)*		2.17 *(1.14–4.11)* P = 0.01	**2.08** *(0.95–4.54)*	1.91 *(0.86–4.27)* P = 0.11
Log10 HIV RNA at baseline (by 1)	1.21 *(0.94–1.57)*			1.04 *(0.74–1.46)*	
CD4 cell count (by 50 cells/mmc)§	0.89 *(0.81–0.98)*		0.92 *(0.84–1.01)* P = 0.06	0.94 *(0.84–1.04)*	
Past ARV regimens (1–5 as reference)					
6*–*8	0.85 (*0.37–1.95)*			0.97 *(0.35–2.67)*	
9*–*12	1.04 *(0.48–2.27)*			0.88 *(0.32–2.41)*	
≥13	0.95 *(0.40–2.23)*			0.60 *(0.18–2.04)*	
ART duration before study entry (by 1 year)	1.00 *(0.95–1.05)*			1.03 *(0.96–1.11)*	
HCV/HBV coinfection (Yes vs No)	0.97 *(0.53–1.80)*			1.04 *(0.47–2.31)*	
Genotipic Sensitivity Score (by 1 point)	0.94 *(0.65–1.36)*			*0.94 (0.59–1.52)*	

ITT  =  intention-to-treat analysis, OT  =  on-treatment analysis, IDU  =  intravenous drug user; ARV  =  antiretroviral; ART  =  antiretroviral therapy; HCV  =  Hepatitis C Virus; HBV  =  Hepatitis B Virus.

* only including selected variables (P<0.15).

§ P<0.05.

Genotypic analysis was obtained for 14 out of 29 failing patients, and RAMS are shown in Table 3.

**Table 3:** Resistance-associated mutations (RAMs) to integrase inhibitors in 14/29 strains from patients with virological failure to a raltegravir-based regimen.

Patients	RAMs
1.	N155H°
2.	N155HN°
3.	N155HN°
4.	N155H, E157Q°
5.	S143C, N155H°
6.	72I, 73V, 140S, 148H, 165I°
7.	72I, 140A, 148R°
8.	72I, 140S, 148H, 165I, 113IV, 119P, 123S, 124T, 127K, 138AE, 154I°
9.	72I, 74M, 151I, 165I§
10.	72I, 74I, 97A, 45V, 88I, 119R, 123S, 124T, 125A, 127K, 143A§
11.	97A, 143R, 163R*
12.	72Î
13.	72Î
14.	72Î

RAL  =  raltegravir, ELV  =  elvitegravir, interpretation from: http://sierra2.stanford.edu/sierra/servlet/JSierra , accessed Feb, 2, 2012

°High level resistance to RAL & ELV,

§ Potential low-level resistance to RAL & ELV,

* High level resistance to RAL but still susceptible to ELV,

ˆ Minor mutation without impact.

### Safety Analysis

**Figure 2:**
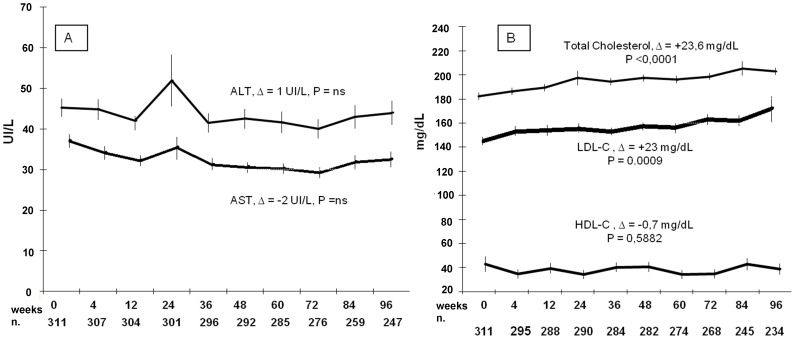
Metabolic impact of raltegravir-based salvage salvage regimens over 96 weeks. In both figures values are presented as mean±standard deviation (SD). The dimension of the cohort at each timepoint is reported below (n.). (2A) AST and ALT slope over 96 weeks, all data caught (even 1 acute hepatitis B), (2B) total and fractionated cholesterol slope.

Nine (3.1%) and 7 (2.1%) patients reached AST and ALT serum levels over grade 2 toxicity respectively, while one and 28 (8.8%) had over grade 2 toxicity of TC and TG serum levels, respectively. Only TC showed an overall increasing trend over time, with a mean increase of +23.6 mg/dL at week 96 (P<0.0001, Figure 2).

The multivariable analysis took into account gender, age, route of HIV transmission, GSS of the backbone therapy, CDC stage C, duration of HIV infection, number of regimen changes, HBV/HCV coinfection, baseline CD4+ T cell counts, HIV-1 RNA, and baseline TG, TC and ALT levels. Only baseline altered ALT (>35 International Units/L) were a significant risk factor of having any level of ALT elevation by week 96 (*P*<0.0001). Duration of HIV infection (*P* = 0.02), hepatitis B or C coinfection (31% versus 60%, *P* = 0.006) and baseline elevated TC (*P*<0.0001) predicted week 96 TC elevation over the National Cholesterol Education Program guidelines [13] optimal level, while for TG the only predictors were male sex (36% versus 18%, *P* = 0.018), and baseline elevated TG (*P*<0.0001). Other adverse events with their CTCAE grading are reported in Table 4.

**Table 4:** Adverse events (n) reported during the observation, classified by CTCAE grade.

Adverse event (>1)*	Grade1	Grade2	Grade3	Grade4	Grade5
ALT Elevation	21	18	9	1§	–
AST Elevation	31	2	6	1§	–
Triglyceride elevation	64	64	28	–	–
Total Cholesterol elevation	157	32	1	–	–
CK Elevation	2	–	1	–	–
Non-Hodgkin’s Lymphoma	–	–	–	1	5
Liver Cirrhosis	26	22	18	15	3
Acute Myocardial Infarction	–	–	2	–	1
Pulmonary Tuberculosis	–	–	–	–	1
Hepatocarcinoma	–	–	–	2	–
Acute psychosis	–	1	–	1	–
Fatigue/Malaise	–	–	2	–	–
Gastrointestinal Intolerance	2	1	1	–	–
Anxiety	–	1	1	–	–
Headache	–	2	–	–	–
Weight Loss	–	2	–	–	–
Flu-like Symptoms	2	1	–	–	–
Myalgia	2	1	–	–	–

§ Acute hepatitis HBV,

* Plus one case of: renal cancer, grade 3 rhabdomyolysis, non-Hodgkin’s lymphoma, coronary heart disease, diabetes, Basedow’s disease, portal hypertension, arterial hypertension, urinary tract infection, fever, acute psychosis, dizziness, flushing, macular rash of skin and glans, gout, cataract, dry skin, asymptomatic hyperuricemia and LDH increase.

### Analysis Stratified by Number of Drugs Composing the Regimen

The whole cohort was stratified according to the number of drugs of which the new regimen was composed: 2 (n = 45, group A), 3 (n = 111, group B), 4 (n = 124, group C), or >4 (n = 40, group D). The clinicians’ decision to combine more or less drugs was oriented by the GSS (mean±SD: 1.60±0.40 in A; 1.88±0.81 in B; 2.11±0.80 in C; and 2.13±0.91 in D) and the baseline CD4+ T-cell count (mean±SD: 367±261 cells/mm^3^ in A; 260±181 cells/mm^3^ in B; 260±189 cells/mm^3^ in C; and 167±160 cells/mm^3^ in D), while the baseline viremia did not influence such trend (mean±SD: 3.9±0.8 plasma HIV-1 RNA log_10_ copies/mL in A; 4.0±1.1 in B; 3.9±1.1 in C; and 4.36 in D). The absolute CD4+ T-cell gain was comparable between arms (+270, +214, +216, and +240 cells/mm^3^, respectively), as was the proportion of subjects maintaining HIV-1 RNA <50 copies/mL at week 96 (Table 5), at least in A, B and C (88.9; 87.3; and 87.9%, respectively), while group D had some more problems related to adherence and to the poor baseline immune status: in fact, only 80% had viral load below 50 copies/mL.

**Table 5:** Baseline and efficacy and safety data of the Regimen Complexity Analysis.

Regimen	2 Drugs (A) N = 45	3 Drugs (B) N = 111	4 Drugs (C) N = 124	>4 Drugs (D) N = 40
**RAMs NA, mean**	6.2	5.4	5.2	7.6
**RAMs NNRTI, mean**	1.5	1.5	1.4	2.7
**RAMs PI, mean**	5.8	8.2	7.5	12.2
**GSS, mean**	1.6	1.9	2.1	2.1
**% HIV RNA <50 copies/mL**	88.9	87.8	87.9	80
* **Δ CD4+ T cells/mm^3^, mean** **(95% CI), P**	+270.2, (134.5–406.0)**P<0.0001**	+214.2 (164.5–267.9)**P<0.0001**	+216.5 (141.5–281.4)**P<0.0001**	+244.1 (170.6–317.7)**P<0.0001**
* **Δ Total Cholesterol, mg/dL, mean** **(95% CI), P**	+17.0 (−3.4 – +38.5)P = 0.10	+26.0 (12.3–39.7)**P = 0.0002**	+18.7 (5.7–31.7)**P = 0.0049**	+34.4 (16.3–66.1)**P = 0.0016**
* **Δ Triglycerides, mg/dL, mean** **(95% CI), P**	−41.9 (−94 – +10.2)P = 0.11	+5.7 (−43.1 – +53)P = 0.82	−14.8 (−48.9 – +19.3)P = 0.39	−3.8 (−69.6 – +45.1)P = 0.69
* **Δ ALT, IU/L, mean (95% CI), P**	−1.4 (−24.8 – +11.4)P = 0.46	−2.2 (−14.1 – +9.8)P = 0,72	−1.4 (−12,7 – +9,8)P = 0,80	+1.1 (−24,2 – +26,3)P = 0,93

RAM  =  resistance-associated mutations; NA  =  nucleoside (nucleotide) analogues; NNRTI  =  non nucleoside reverse transcriptase inhibitor; PI  =  protease inhibitor; GSS 0 genotypic sensitivity score; ALT =  alanine aminotranferase, 95% CI  =  Confidence Interval.

* Δ values are mean differences between baseline and w 96.

### Analysis of Companion Drugs’ Contribution to Viral Suppression

In group A 8 subjects (17,8%) introduced at least a new drug class together with RAL, while groups B, C and D this occurred to 33 (29,7%), 29 (20,6%) and 10 (25%), respectively.

The main accompanying drugs were darunavir (53% in arm A, dual therapy 50% in B, 67% in C, and 86% in D), lamivudine or emtricitabine (55% in B, 94% in C, and 97% in D), tenofovir (12% in B, 78% in C, and 80% in D), etravirine (26% in B, 16% in C, and 63% in D) and maraviroc (20% in B, 4% in C, and 9% in D). Overall, the accompanying drugs were generally recycled as the least damaged in long treatment histories.

In arm A, 10/19 subjects receiving boosted darunavir had RAMs conferring significant loss of sensitivity towards this drug at baseline, in arm B the proportion was 35/58, in arm C 40/83 and in arm D 31/36.

Lamivudine/emtricitabine RAMs were present in 42/54 3TC/FTC recipients in arm B, 92/113 in arm C and 35/39 in arm D, tenofovir RAMs in tenofovir-treated patients were 2/12 in arm B, 55/95 in arm C and 25/33 in arm D, and finally etravirine RAMs in etravirine-treated subjects were in 0/1 in arm A, 12/28 in arm B, 7/21 in arm C and 16/30 in arm D.

Maraviroc sensitivity was based upon prediction by Enhanced Trofile and the drug could not be administered without this result, so it is considered to be active at baseline in all cases.

Finally, we analyzed the contribution of darunavir to virologic suppression and CD4+ T-cell gain. Darunavir-containing regimens (n = 203) achieved a mean CD4 increase of +223/mm^3^ with 7,3% virologic failures, while in darunavir-sparing regimens (n = 117) CD4+ T-cells increased by +236/mm^3^ on average with a 10% virologic failure rate.

## Discussion

To our knowledge, this is the largest observational cohort of salvage therapy with raltegravir and the longest follow-up reported to date.

The main comparison that can be made is with the BENCHMRK study data at 96 weeks [2]. Despite data being collected from observational cohorts, the proportion of subjects with HIV RNA <50 copies/mL at week 96 is remarkably higher in our study, as well as the CD4+ T-cell gain. From a clinical point of view we observed a comparable proportion of AIDS events (2,2%), while, aside a comparable proportion of AIDS-related deaths, we had 5 (1,5%) non AIDS-related deaths. A larger proportion of subjects was on concomitant darunavir therapy as compared to the BENCHMRK study, however this does not seem to have yielded better results. In both studies the GSS and the number of active drugs were not associated with different virologic outcome.

The ANRS 139 TRIO study showed very good results in a completely different setting, where a brand new regimen composed of etravirine, boosted darunavir and raltegravir acted in the setting of an optimal GSS ( = 3) [14]. The overall virologic suppression rate is comparable to that of the SALIR group, and the CD4+ T-cell increase smaller (+150/mm^3^ vs + 227/mm^3^).

The main results of the present study are indeed the immunologic gain and the protection from progression to AIDS after 96 weeks of salvage therapy in a consistent cohort (n = 320), representative of numerous centers from Italy. Also, the liver and metabolic toxicity that we observed in our cohort is quite mild and liver enzymes’ elevations were mainly seen in HCV-infected subjects, as reported by Vispo et al [15].

The progressive increase in total cholesterol is confirmed in the pilot SHIELD trial (n = 35) [16], but not in the SPIRAL study (n = 273) [17], in which previous boosted protease inhibitor-based regimens were simplified to raltegravir. The increase of cholesterol levels could be explained considering that uncontrolled HIV infection results in substantial decreases in serum TC. Effective antiretroviral therapy is associated with increases in cholesterol: this represents a return to pre-infection serum lipid levels after accounting for expected age-related changes [18]. In our data, confirming a previous study, hepatitis coinfection contains the cholesterol increase [19].

Furthermore, confirming a previous smaller experience [20], having only one drug (a non-nucleoside reverse transcriptase inhibitor or a boosted protease inhibitor) in the backbone was associated with a high proportion of suppression of HIV-1 replication (88.9%) at week 96 and with a non significant increase in total cholesterol.

In treatment-experienced subjects, a raltegravir-including salvage ART achieved and maintained high levels of virologic suppression, with few virologic failures, mostly accompanied by the emergence of resistance-associated mutations. The impact on metabolic and liver function was mild, also because of the presence of relatively low-impact backbone regimens, mostly containing darunavir and/or etravirine and/or tenofovir/emtricitabine. The proportion of deaths could be explained with the advanced stage of HIV-1 infection of the involved subjects.

Considering the highly advanced HIV-infection of our cohort, we observed few serious adverse events and deaths; tumors were rare; no other new AIDS-defining events were recorded. The SALIR study provides a consistent glance on safety and efficacy of a raltegravir-based salvage therapy in the long run in a “real world” clinical setting.
